# Gelatin Methacrylate Hydrogel for Tissue Engineering Applications—A Review on Material Modifications

**DOI:** 10.3390/ph15020171

**Published:** 2022-01-29

**Authors:** Sasinan Bupphathong, Carlos Quiroz, Wei Huang, Pei-Feng Chung, Hsuan-Ya Tao, Chih-Hsin Lin

**Affiliations:** 1 Graduate Institute of Nanomedicine and Medical Engineering, Taipei Medical University, Taipei 110, Taiwan; kningsnb15@tmu.edu.tw (S.B.); b812107008@tmu.edu.tw (H.-Y.T.); 2 International Ph.D. Program in Biomedical Engineering, College of Biomedical Engineering, Taipei Medical University, Taipei 110, Taiwan; d845109001@tmu.edu.tw; 3 Department of Orthodontics, Rutgers School of Dental Medicine, Newark, NJ 07103, USA; wei.huang.ortho@rutgers.edu; 4 School of Biomedical Engineering, College of Biomedical Engineering, Taipei Medical University, Taipei 110, Taiwan; b812107004@tmu.edu.tw

**Keywords:** GelMA, hydrogel, biomaterials, material modifications, tissue engineering, vascularization

## Abstract

To recreate or substitute tissue in vivo is a complicated endeavor that requires biomaterials that can mimic the natural tissue environment. Gelatin methacrylate (GelMA) is created through covalent bonding of naturally derived polymer gelatin and methacrylic groups. Due to its biocompatibility, GelMA receives a lot of attention in the tissue engineering research field. Additionally, GelMA has versatile physical properties that allow a broad range of modifications to enhance the interaction between the material and the cells. In this review, we look at recent modifications of GelMA with naturally derived polymers, nanomaterials, and growth factors, focusing on recent developments for vascular tissue engineering and wound healing applications. Compared to polymers and nanoparticles, the modifications that embed growth factors show better mechanical properties and better cell migration, stimulating vascular development and a structure comparable to the natural-extracellular matrix.

## 1. Introduction

It has been almost three decades since Cohen et al. first suggested that tissue fabrication may offer improved functions compared to organ transplants [1]. At the same time, Robert Langer and Joseph Vacanti introduced tissue engineering as an interdisciplinary field that merges engineering principles and life sciences to develop biological substitutes to restore, maintain, or improve tissue function [2]. Since then, most of the studies in the field have followed one of three different approaches—using cells or cell substitutes as replacement parts, using acellular biomaterials capable of inducing tissue regeneration, or using a combination of both with a temporary scaffold allowing the cells to reorganize and produce their own extracellular matrix [3].

Over the years, these approaches have improved how we prepare tissue culture and introduced multiple microfabrication processes focused on the cell microenvironment. Organizing the environment allows cells to develop their shapes and signaling networks, resembling their natural behaviors [4]. With the current technology, synthetic tissue still cannot completely replicate the anatomy and physiology of natural tissue. As pointed out by Camci-unal et al. [5], the lack of a three-dimensional biomimetic environment generates stress to the cells during their development. This leads to a high number of deaths and unviable tissue for cardiac tissue engineering. Similarly, attempts to treat spinal cord injury lack adequate supports that allow neuronal regeneration end in unsuccessful attempts as treatment [6]. Additionally, it is still not possible to form a defined vascular network in vascular tissue engineering. Therefore, more studies are needed to find materials with high enough cell binding ability and good mechanical properties to support the cells adequately.

**Scheme 1:**
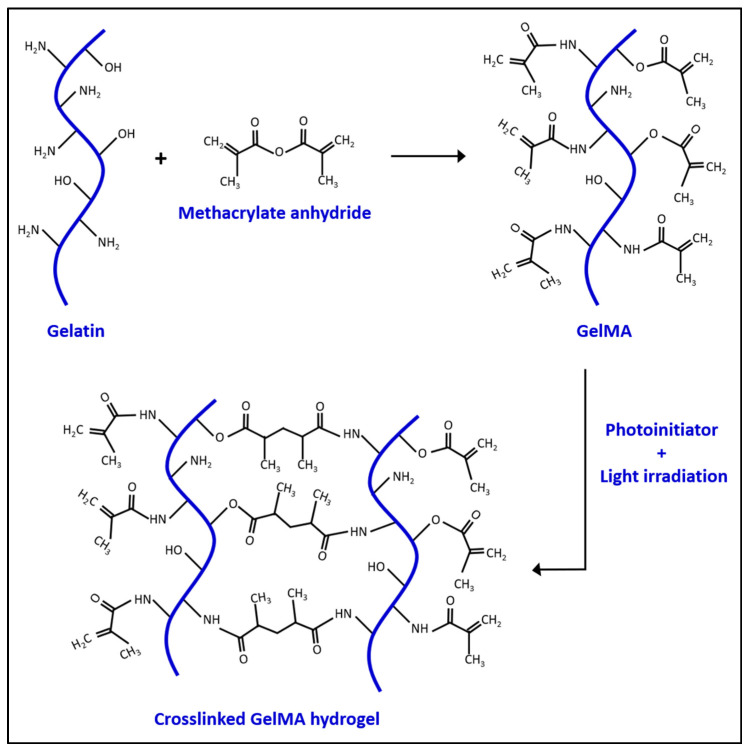
The chemical reaction between gelatin and methacrylate anhydride to produce GelMA and crosslinked GelMA hydrogel.

**Figure 1:**
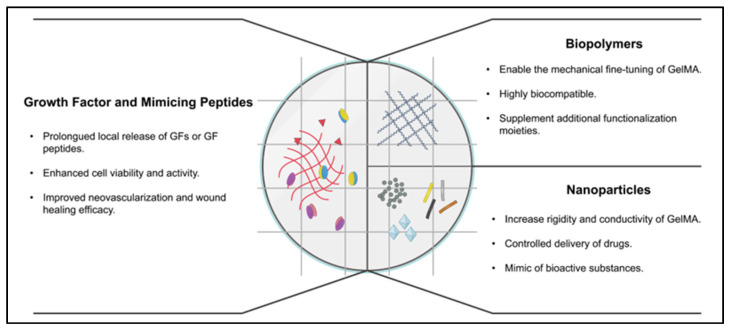
Different modifications of GelMA hydrogel and their benefits.

In the 1990s, Koepff et al. developed gelatin methacrylate (GelMA) by modifying the reactive side groups of gelatin using glycidyl methacrylate [7]. Gelatin is obtained from the denatured collagen, a naturally made protein found in the extracellular matrix [8]. This makes the material biocompatible and biodegradable for cell growth in vitro [9]. Gelatin itself can form a gelatinous structure, but has a relatively low melting temperature (31.7–34.2 °C) which can be a limitation for its use under physiological conditions [10]. Therefore, adding other components to stabilize the gel is needed for in vivo applications [11,12]. In 2000, Van Den Bulcke et al. found that in the presence of methacrylate, GelMA can be crosslinked via photo-polymerization. The crosslinking of GelMA leads to hydrogel formation, as visualized in Scheme 1. Additionally, a series of different gels can also be obtained by controlling the production parameters. This makes GelMA an attractive and inexpensive material for biological applications with a higher melting point than gelatin alone [13]. GelMA can be manufactured through several methods, such as soft lithography [14], electrospray [15], three-dimensional printing [16], microfluidic chip [17], and emulsion [18]. Each method produces different shapes of GelMA for different purposes. For example, Choi et al. used an emulsion technique to generate GelMA microdroplets for neovascularization in mouse models [15]. The purpose of tissue engineering is to build a desirable substrate microenvironment to promote cell growth, differentiation, migration, and function [19]. The stiffness of the cell culture material plays a crucial role in cell behaviors. For instance, mesenchymal stem cells (MSC) develop into osteoblasts when grown on a relatively hard substrate (~40 kPa), but neuroblasts on a softer substrate (~1 kPa) [20]. The porosity of hydrogel substrate is also a key factor as a larger pore size provides an easier path for cell migrations [21]. Generally, the stiffness and porosity of the GelMA hydrogel can be controlled by tuning the hydrogel concentration, degree of functionalization, UV intensity, and additive supplementation [13,21]. The internal polymer network structure of the hydrogel also allows GelMA to accumulate other molecules, such as drugs, and slowly release them out to the environment over time [22]. Additionally, the molecular structure of gelatin makes it possible to covalently functionalize other molecules onto the gelatin to enhance its tissue engineering capabilities, as shown in Figure 1.

This paper reviews the latest functionalization of GelMA through vascular growth factors, nanomaterials, and polymer materials in vascular tissue engineering and wound healing applications. The summary of GelMA with modifiers is shown in Table 1. Biomedical applications in neural tissues, bones, vasculature, muscles, and skin are included in this review. We also reveal what remains unknown and propose where the future lies for the field.

## 2. Vascularization Growth Factors and GelMA

Blood is the main supplier of nutrients and oxygen to cells and tissues in the mammalian body. In addition, cells discard their metabolic wastes into the bloodstream. Thus, vasculature plays an essential role in successful tissue engineering [75]. As summarized by Chung and Shum-Tim [76], the essential angiogenic growth factors are vascular endothelial growth factors (VEGF), fibroblast growth factors (FGF), platelet-derived growth factors (PDGF), and transforming growth factors beta (TGF-β). Each of these growth factors facilitates angiogenesis in its own way, which will be discussed further below.

To achieve a blood vessel network for an implant that is adequate to support the tissue, growth factor concentration and exposure duration/frequency are important factors [77]. In the clinical investigation of VEGF in ischemic patients for vascular angiogenesis, administration of recombinant human (rh) VEGF protein is required at multiple time points during the experiment, and only the high dosage group (1 mg per 50 kg body weight as an initial injection dose) showed improvement in angina [78]. Thus, biocompatible materials can be utilized to reduce the injection frequency and control the released amount of growth factor during the treatment phase.

To date, VEGF-GelMA is the most commonly used growth factor-modified GelMA. TGF-β-conjugated GelMA has also been reported by a few studies. Thus, many opportunities remain further to investigate other growth factors’ effects on GelMA.

### 2.1. VEGF

The VEGF protein family consists of five members: VEGF-A, VEGF-B, VEGF-C, VEGF-D, and PlGF (placental growth factor) [79,80]. VEGF-A plays a crucial role in both vasculogenesis and angiogenesis, especially VEGF-A_165_ [81]. The receptors of VEGF-A are VEGF receptor 1 (VEGFR1) and VEGF receptor 2 (VEGFR2), which modulate the endothelial homeostasis and the angiogenic process [82]. In comparison to VEGFR1, VEGFR2 shows more roles in angiogenesis as it controls the growth, differentiation, and migration of endothelial cells to develop tube-like structures [83].

To supply VEGF in the in vivo models, conventional methods include bolus injections and intravenous injections [78,84]. The drawback of these methods is that they require multiple VEGF injections, which take at least 20 min to sometimes hours per patient for one injection [78]. In addition, Waller et al. reported that intravenous injection of elastin-like polypeptide-linked VEGF-A_121_ (ELP-VEGF) at a high dose in mice could cause hypotension, GFR increase, renal microvascular density reduction, and tumor vascularization [85]. Therefore, sustained and localized release of drugs or bioactive molecules from hydrogels can help to prevent systemic toxicity and enhance wound healing [86,87].

**Figure 2:**
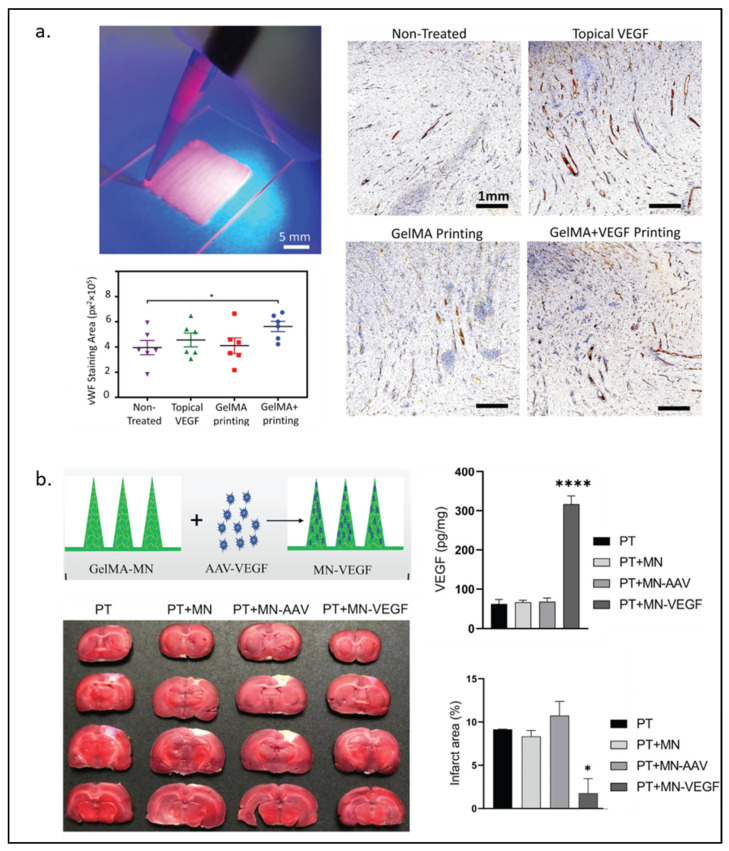
VEGF-modified GelMA hydrogel. (**a**) Handheld printing using VEGF-modified GelMA hydrogel as bioink for wound healing (left) and immunostained for von Willebrand Factor under an optical microscope (right) with quantitative results of wound bed angiogenesis by measurement of vWF signal in different wounds (n = 6). The data is presented as mean ± standard error of mean (SEM). Comparison of the different groups was performed using a student’s T test and * represents *p* < 0.0005 (bottom left). (**b**) Schematic illustration of GelMA-microneedles loaded with AAV-VEGF and the results of cerebral ischemia treatment in rats among different groups treated. (Photothrombotic (PT), microneedle (MN), adeno-associated virus (AAV), vascular endothelial growth factor loaded microneedle (MN-VEGF)). Statistical analysis was performed using a two-sided Student’s t-test or one-way analysis of variance (ANOVA). of the infarct area (upper right) and the protein levels of VEGF in the brain 3 weeks after MN implantation (lower right). The data are expressed as the mean ± SD. * *p* < 0.05 and **** *p* < 0.0001. Reproduced (adapted) with permission from Ref. [23]. 2022 © Biomaterials, and from Ref. [27]. 2021 © Journal of Controlled Release.

In VEGF-loaded GelMA, it may be adequate to have GelMA physically trap VEGF without covalent bonding between them. Recently, Nuutila and Samandari et al. [23] invented an in situ hand-held VEGF-GelMA printer. VEGF was not found to be covalently bonded to GelMA. Yet, VEGF was shown to be continuously released from the gel for at least 7 days and promoted HUVECs migration in the scratch assay. Furthermore, the VEGF-GelMA printed on porcine wounds showed the highest level of angiogenesis at the wound bed among all experimental groups, including a group with topical delivery of VEGF method as observed in Figure 2a. Without the covalent bond of VEGF and the gel, Wang and Chen et al. [88] also demonstrated that the non-covalently bonded VEGF on the BMP2-GelMA can promote osteogenesis better than BMP2-GelMA alone.

In addition to the natural VEGFs, the VEGF mimetic peptide called QK is also widely used for GelMA modification. QK mimics the VEGF 17–25 helix region, which can activate VEGFRs and enable the downstream pathways [88]. Parthiban et al. [24] used the acrylate QK to photo-crosslink GelMA. They showed high levels of CD34, Ang2, and vWF expression by HUVECs cultured on QK-GelMA at day 5, indicating that the hydrogel was capable of promoting vascularization. Similarly, John et al. [25] modified QK with octenyl alanine at the *N* terminus end to help photo-link QK with GelMA. After 7 days of incubation of HUVECs on QK-GelMA, dense microvascular networks with interconnections could be seen through immunostaining and confocal microscopy. Similar to the full-length VEGF, the QK peptide was also loaded to GelMA without covalent bonding by Jang et al. [26], and the result demonstrated that HUVECs formed the tubular structure in the QK-trapped GelMA. Furthermore, this 3D-printed QK-GelMA could accelerate wound healing in porcine models.

Instead of supplying VEGF or QK through GelMA, Lie et al. [27] fabricated GelMA microneedles loaded with an adeno-associated virus vector expressing VEGF (AVV-VEGF) for angiogenesis and functional recovery after stroke in mice. Their result showed that mouse brains were able to express VEGF, decrease the infarct area, and recover the motor functions, as shown in Figure 2b.

### 2.2. TGF-β

The TGF-β family consists of TGF-β1, TGF-β2, and TGF-β3. These cytokines play an essential role in both healthy and pathological stages of tissues, such as maintaining the homeostasis of the cell cycle in normal tissue or inducing inflammation and angiogenesis in the wound healing process [89]. TGF-β regulates cell behavior through the canonical SMAD pathway or non-canonical signaling (e.g., MAPKs and PI3K pathways) [90].

In tissue engineering applications, TGF-β was widely used to supplement hydrogel materials to enhance therapeutic effects, such as cartilage regeneration and the aneurysm and pseudoaneurysm treatment [91,92,93]. The loading method of TGF-β onto GelMA can be through either physical network trapping or chemical bonding. In a study reported by Cao et al. [28], TGF-β was preloaded to GelMA before the polymerization process to allow its trapping within the GelMA network. This TGF-β-GelMA, together with a 3D-printed poly(ε-caprolactone) as a base to enhance its durability, demonstrated the best cartilage regeneration effects in their in vivo experiments.

In the native extracellular matrix, sulfated glycosaminoglycans (GAG), such as heparin and heparan sulfate, are known to be able to bind to many soluble growth factors and further mediate cellular activities [94]. For example, it has been reported that heparin-binding TGF-β3 can enhance neocartilage formation [95]. Thus, in order to bio-functionalize their materials, many research groups were inspired by the protein–heparin binding through the sulfated GAG [96,97,98]. Recently, Wang et al. [29] used alginate sulfate, a sulfated GAG (sGAG) mimic, to functionalize a GelMA hydrogel system to facilitate the bioprinting of cartilaginous tissues. TGF-β3, loaded into the hydrogel with an equivalent of a 6-week dose (240 ng/construct) prior to crosslinking, was found to have a continuous release from the sulfated-alginate-GelMA over a 21-day course. Meanwhile, the release of TGF-β3 plateaued on day 7 in the alginate-GelMA and GelMA controls. Histological analysis demonstrated a higher level of sGAG and type II collagen in the TGF-β3-loaded alginate-GelMA mouse samples compared to the non-loaded alginate-GelMA controls.

**Figure 3:**
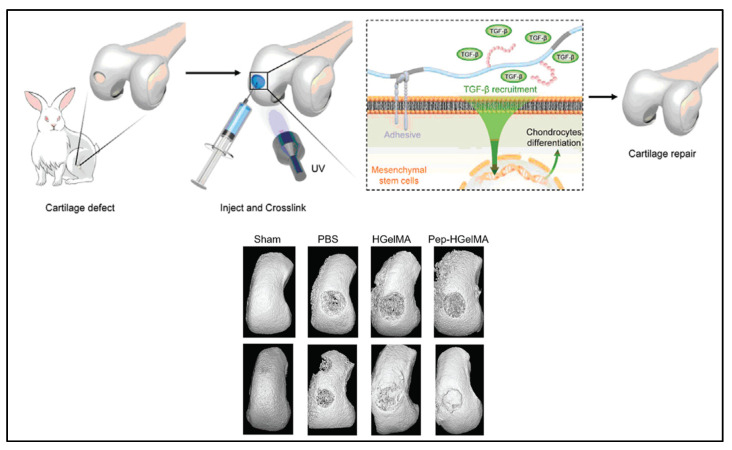
A schematic illustration of a novel photo-crosslinked GelMA hydrogel and HSNGLPL-MA recruits endogenous TGF-β1 and enhances cartilage regeneration. A 3D reconstruction of micro-computed scans after cartilage repair with GelMA modified with TGF-β1 affine peptides in rabbits. Reproduced (adapted) with permission from Ref. [30]. 2021 © Smart Materials in Medicine.

The use of TGF-β1-affinity peptide (HSNGLPL) to increase the sequestration of TGF-β1 by a substrate has been reported by several groups [99,100]. Ju et al. [30] modified the GelMA hydrogel with methacrylated-HSNGLPL and investigated its effect on chondrogenesis and cartilage regeneration. Crosslinked HSNGLPL-GelMA hydrogel was found to have increased material storage modulus and ability to recruit TGF-β1 and induce mesenchymal stem cell differentiation to chondrocytes in vitro. As in an in vivo knee joint injury rabbit model shown in Figure 3, implanted HSNGLPL-GelMA hydrogel showed the highest level of cartilage regeneration compared to the GelMA hydrogel without the HSNGLPL group.

### 2.3. Growth Factor in Platelet Lysate

Platelets contain many growth factors, including VEGF, TGF-β, PDGF, IGF, EGF, and FGF [101,102]. Barsotti and Losi et al. [103] reported that platelet lysate could promote cell viability and migration and activate intracellular pathways. Platelet-rich plasma (PRP) can be an economical and efficient source when multiple growth factors are needed in the application [31].

Platelet lysates are usually incorporated in GelMA via the physical capture manner in tissue engineering. For instance, human platelet lysate can be preloaded to GelMA bioink, and the structure can be 3D printed [32]. Other materials can also be added to the mixture to modify the hydrogel’s mechanical properties. Lu and Li et al. [104] loaded the PRP to the GelMA-silk fibrinogen methacrylate (GelMA-SFMA) hydrogel and investigated its effect in a mouse wound healing model. The result suggested multiple abilities of the PRP-GelMA-SFMA, including promoting wound healing, collagen deposition, angiogenesis, and nerve regeneration. Zhang et al. [31] supplemented GelMA-nano clay with platelet lysate and found that, interestingly, platelet lysate-loaded GelMA could similarly enhance neovascularization both with and without nano clay.

## 3. Natural-Derived Polymer and GelMA

Mechanical properties of GelMA can be tuned to the desired condition in many different ways, from a simple method of varying the gel concentration to a more complicated one of co-polymerization. Currently, numerous types of polymers, both naturally derived and synthetic, have been used to couple with GelMA. This section focuses on the natural polymers that can co-polymerize with GelMA.

### 3.1. Hyaluronic Acid

**Figure 4:**
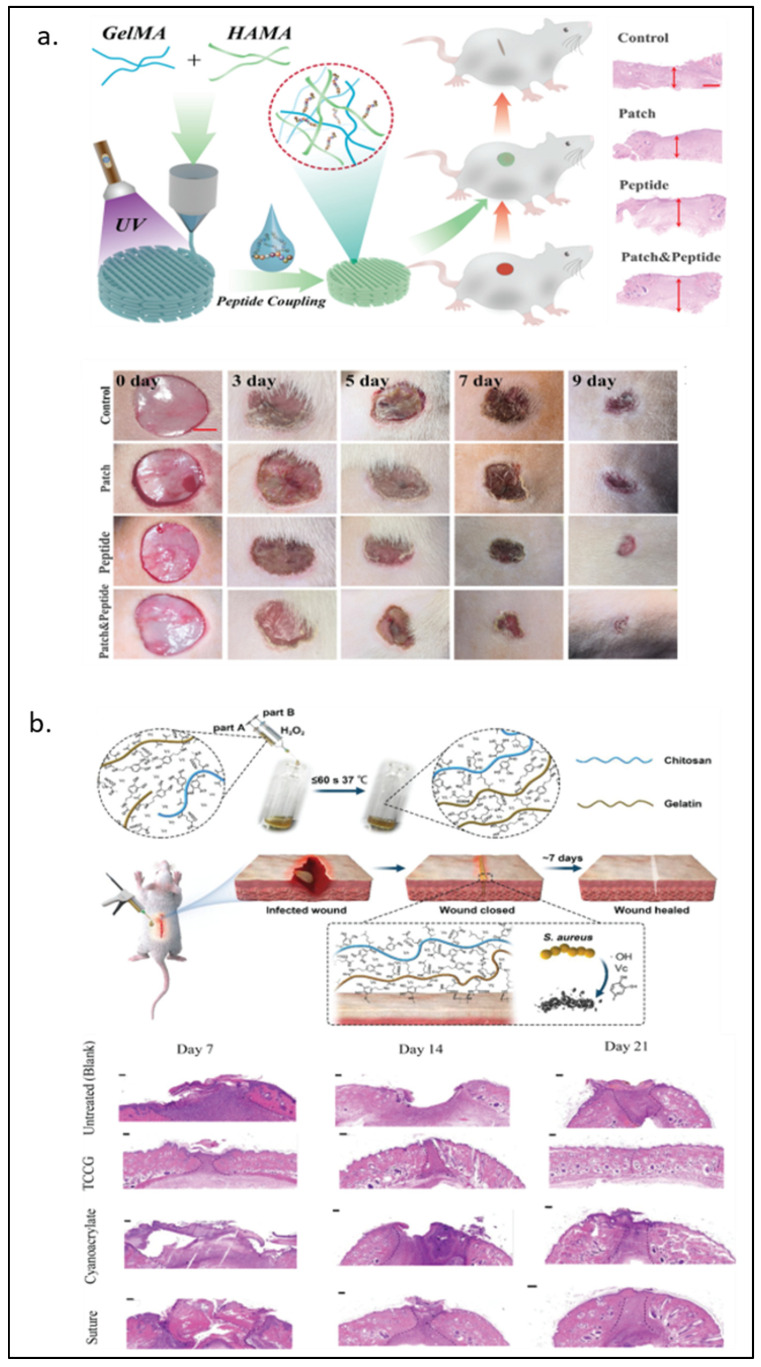
GelMA modified with naturally derived polymers. (**a**) Hyaluronic acid-modified GelMA for wound therapy from 0–9 days wounds and photographs of wound healing progress. (**b**) Schematic diagram of GelMA/chitosan wound adhesive doped with dopamine with antibacterial properties and H&E staining on days 7, 14, and 21 observed under an optical microscope. Reproduced (adapted) with permission from Ref. [35]. 2022 © Materials Today Bio, and Ref. [40]. 2020 © Carbohydrate polymers.

Hyaluronic acid, a type of Glycosaminoglycan (GAG), is an important component of the extracellular matrix (ECM) [105] that has been incorporated into hydrogels in tissue engineering material developments [106,107]. In 2017, Eke and Mangir et al. [33] developed the photo-cross-linkable GelMA and methacrylated hyaluronic acid (HAMA) cell-laden hydrogel for angiogenesis and wound healing purposes. Lee and Park et al. [34] used 3D printing to construct a more complicated structure of a rabbit larynx from GelMA and HAMA. The GelMA-HAMA structure remained stable three weeks after in vivo implantation and induced increased type II collagen expression over time. Recently, Guan et al. [35] covalently attached the novel pro-angiogenic peptide, QHREDGS, onto 3D-printed GelMA-HAMA patches. These patches significantly improved the migration of HDF cells and the tube length formed by HUVECs. As shown in Figure 4a, animal wound healing models also showed the best recovery percentage with the lowest proinflammatory factors expression in the peptide-loaded hydrogel group.

### 3.2. Chitosan

Chitosan is a product of deacetylation of chitin—an amino-polysaccharide polymer found in shrimp and crab shells [108]. Due to its similarity to GAGs in structure and characteristics [109], along with its biocompatibility, biodegradability, and antimicrobial [108,110], chitosan has been a popular biomaterial for biomedical applications such as drug carriers and tissue engineering [111,112]. The addition of chitosan has been shown to improve the stability of GelMA. Suo and Zhang et al. [36] took advantage of the pH-dependent solubility of chitosan to manipulate the chitosan network formation. The group found that Young’s modulus of 10% GelMA around 4 kPa became around 60 kPa when 2% chitosan was added, and chitosan formed the interpenetrating network (IPN). Chitosan crosslinking happens in the basified environment, in which the electrostatic repulsion between cationic residues on the chitosan is reduced, but hydrophobicity and hydrogen bonding between each chitosan chain is intensified [113]. Without modification, chitosan was used with GelMA as a cell carrier in the 3D co-culturing system [37]. With modifications, chitosan has been shown to have more functionalization capabilities. For instance, Luo et al. [38] modified chitosan to be methacrylated hydroxyl butyl chitosan (MHBC), a thermo-responsive compound, and photo-crosslinked it with GelMA. Zhang et al. [39] coupled cysteine, a thiol group source, on chitosan to allow the Mg^2+^ loading in thiolated chitosan/GelMA. The results showed that the Mg^2+^ loaded hydrogel promoted osteogenesis and angiogenesis in both in vitro and in vivo models. Methacrylated-chitosan and GelMA were further modified with protocatechuic acid and dopamine to make an adhesive wound-closure biomaterial, where H_2_O_2_ and ascorbic acid could polymerize the gel within seconds [40]. In Figure 4b, bacteria-infected wounds in animal models showed complete wound closure at day 21 after the chitosan-GelMA treatment, while the small gap at the wound remained visible with the conventional suture treatment.

### 3.3. Alginate

**Figure 5:**
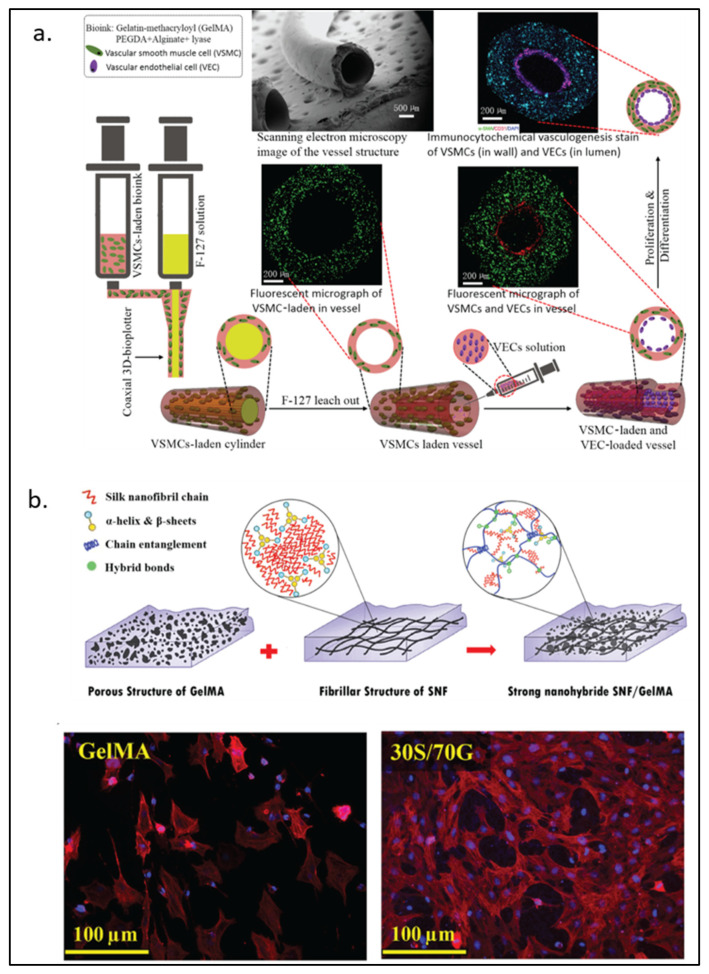
GelMA modified with naturally derived polymers. (**a**) Schematic representation of 3D-printed biomimetic blood vessels with alginate modified-GelMA. (**b**) Silk fibroin-modified GelMA for corneal regeneration with fluorescence imaging DAPI (blue) and rhodamine-phalloidin (red) after 5 days of culture. Reproduced (adapted) with permission from Ref. [42]. 2020 © ACS Applied Materials and Interphases; and Ref. [48]. 2021 © Material Science & Engineering C.

Alginate, a natural block-copolymer from brown algae, consists of (1,4)-linked β-d-mannuronate and α-l-guluronate residues. Although lacking the intrinsic mammalian cell adhesion motif (e.g., RGD motif), alginate remains a primary choice of material for many biomedical applications because of its biocompatibility and low cost [114]. As GelMA can provide the RGD motif for cells to attach, alginate can be functionalized by GelMA into a cell attachable substrate. Conversely, alginate can also be used to interpenetrate GelMA to enhance its mechanical property, be a sacrificial part, or provide functionalized moieties for the materials. Ansari et al. [41] reported the use of a gingival mesenchymal stem cells (GMSCs)-laden alginate-GelMA platform for a wound healing application. More complex structures such as small blood vessels have also been formed from the alginate-GelMA hydrogel system. As shown in Figure 5a, Zhou et al. [42] employed co-axial 3D printing to build a small hollow tubular hydrogel structure with an inner diameter of around 1 mm. The tube wall contained GelMA, polyethylene-(glycol) diacrylate, alginate, lyase, and vascular smooth muscle cells. In this model, lyase gradually removed alginate in the structure so that more space within the hydrogel could be freed for cells to proliferate better. In addition to modifying the mechanical property of a hydrogel system, alginate can also be chemically or biochemically modified for an improved wound healing process. As discussed earlier, sulfated alginate has been shown to absorb and bind endogenous TGF-β3 via electrostatic forces, which provide better support for chondrogenesis both in vitro and in vivo [29]. In an antibacterial wound healing study, Yao et al. [43] used oxidized sodium alginate to form Schiff-base bonds with GelMA. They showed improved material storage modulus and slow release of antibiotics at a physiological condition.

### 3.4. Silk Fibroin

The polypeptide of fibroin can be found at the inner core of the silk building block. Due to its excellent mechanical strength, biocompatibility, enzymatic degradability, and biomolecule durability, silk fibroin has become an attractive material in biomedical research [115].

The IPN of silk fibroin in GelMA can strengthen the modulus and stiffness of the hydrogel [45]. In addition, silk fibroin particles have also been used to increase the viscosity of pre-crosslink GelMA, which helps prevent cell sedimentation during the cell-laden 3D-printing process [46]. The mechanical properties of silk fibroin-GelMA are tunable, depending on the ratio between the two components. For load-bearing soft tissue engineering, Xiao and Li et al. [47] found that the 9:1 silk fibroin to GelMA ratio could increase the compress modulus value to a level higher than that of silk fibroin. The group also demonstrated the biocompatibility of silk fibroin-GelMA with its use as an osteoblast adhesion surface and a cell-laden scaffold. Farasatkia et al. [48] invented the transparent silk fibroin-GelMA material, where the optimal ratio between the two polymers was found to be 30% silk fibroin and 70% GelMA as shown in Figure 5b. This ratio gave the hydrogel good transparency and cell proliferation support. Farasatkia and his team further developed a more suitable material for corneal regeneration by applying micro-patterns to silk fibroin-GelMA to mimic the stroma ECM cell growth orientation together with an alginate layer to provide an adhesive property [49].

## 4. Nanomaterials and GelMA

A biocompatible artificial scaffold offers an ideal strategy for tissue engineering. Although GelMA provides high versatility and exceptional conditions for cells, by itself, it cannot provide a rigid enough structure needed for bone development or the electromechanical property required by skeletal muscle, cardiac and neural cells. Therefore, numerous hybrid hydrogels modified by nanomaterials have been developed to cultivate certain cells in their specific environment to stimulate their functions.

### 4.1. Carbon-Based Nanomaterials

Hybrid carbon-based materials such as graphene oxide (GO), carbon nanotubes (CNT), or nano-diamonds (ND) are able to provide many mechanical and/or electrical properties needed for the hydrogels [116]. Due to their chemical structures, carbon-based materials can increase the strength and electrical conductivity while retaining similar degradability, porosity, and cell dispersion offered by GelMA. Another advantage of carbon-based materials is that they can be functionalized with a series of particles. GO is a two-dimensional sheet of carbon atoms that has been functionalized with titanium and crosslinked with GelMA hydrogel under UV radiation and was used as a coating for orthopedic implants. This modification works as a zinc reservoir, which is favorable for osteoblast adhesion and spreading. In addition, compared to titanium, it has also demonstrated an improved antibacterial effect against *P. aeruginosa* and *S. aureus*, pathogens importantly related to implant-associated infections in orthopedic surgeries [117].

**Figure 6:**
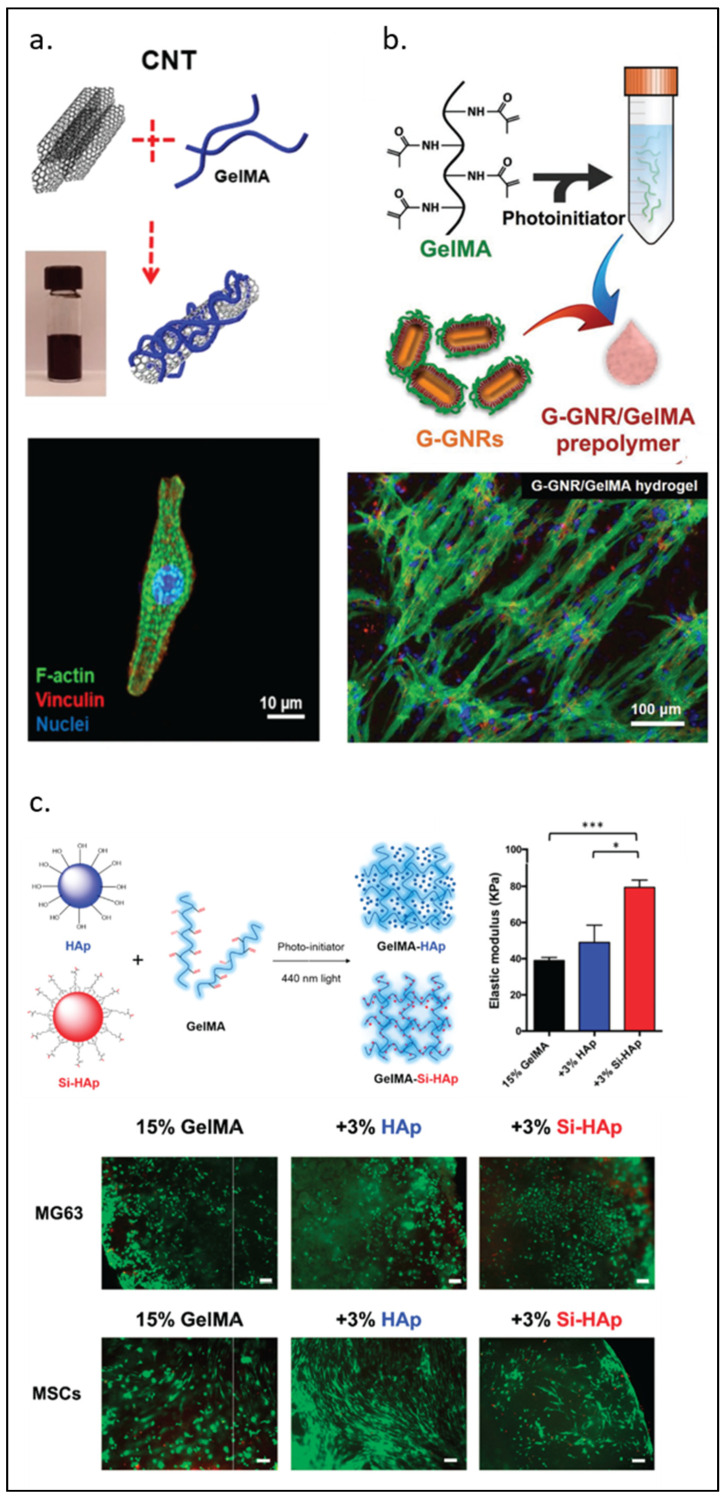
GelMA modified by nanomaterials. (**a**) Carbon nanotubes. (**b**) Gold nanorods for cardiomyocyte constructs. (**c**) Biofabrication of silanized hydroxyapatite modified cell-laden GelMA and MG63 cells and human mesenchymal stem cells (MSCs) encapsulation by GelMA composite (green, calcein AM; red, ethidium homodimer-1). Scale bar, 100 µm. Elastic modulus of GelMA-HAp and GelMA-Si-HAp hydrogels data is presented as mean ± standard deviation. * *p* < 0.05, *** *p* < 0.001 upon one-way ANOVA analysis (upper right). Reproduced (adapted) with permission from Ref. [50]. 2019 © ACS Nano. Ref [57]. 2017 © Advanced Functional Materials Smart Materials in Medicine. And Ref. [63]. 2021 © Polymers.

CNTs have been shown to offer different properties depending on their diameter, length, and sometimes single or multi-walled structure. Junmin Lee et al. [50] developed scaffolds using carbon nanotube hybrids aiming to improve cardiac cell organization and proliferation. This study compared rGO, GO, and CNT modified GelMA revealing that these hybrids have different effects on the spreading and maturation of cardiomyocytes, as observed in Figure 6a. The CNT-GelMA hybrid scaffold offered better mechanical properties, smaller pore size, and better uniformity (less surface roughness), which might be the reason behind the improvement in the differentiation of cardiomyocytes. Other researchers also reduced GO (rGO)/GelMA, improving the conductivity and re-orientation of cardiomyocytes, which is a critical setting to improve contractile function [52]. NDs are a type of octahedral carbon-based material that has shown reduced toxicity and biodistribution compared to other materials in this group. They can be functionalized with multiple other moieties to modify their biological properties. When combined with GelMA, NDs can exert their properties to a wide variety of applications [55]. Many studies on carbon-based modified GelMA have focused on the recreation of the microenvironment for the bone [56], cardiac [51,53], nerve [54,118], and skin [119,120] tissues, as well as drug delivery and encapsulation.

### 4.2. Metal-Based Nanomaterials

Although carbon is a great conductor that can improve GelMA’s conductivity and mechanical strength, there are concerns regarding toxicity and biodegradability. To address these concerns, researchers have explored metal-based modifications as an alternative. Gold or silver nanoparticles are also able to provide strength, conductivity, or antimicrobial properties to the gel [121].

As observed in previous studies, cell reorganization is a determining factor in improving the tissue’s functionality [122]. This is particularly true for myocardial cells that follow a defined structure, coordinated movement, and conductivity to achieve heart contraction. Zhu et al. [57] designed a modified gold nanocomposite bioink for 3D bioprinting of cardiac tissue that improved their function and electrical propagation, as observed in Figure 6b. Compared to the GelMA/alginate bioink, cells in GelMA/gold bioink were better spread after 5 days and elongated with higher contractile frequency after 12 days of culture. Navaei et al. [58,59] compared silica nanoparticles (non-conductive) and gold nanorods (conductive) for modifying GelMA scaffolds. They found similar retention, cell–cell coupling, protein expression, and excitation threshold between conductive and non-conductive scaffolds. This suggests that the structure stiffness carries a prominent role in cardiac tissue engineering; similar results were observed in gold nanowires [58,59]. Recently, silver nanoparticles modified GelMA has received increased interest because of its antimicrobial capabilities. Ou et al. [60] designed a nanosilver/halloysite nanotube/GelMA hybrid hydrogel to enhance bone regeneration. Their in vivo studies showed that the addition of silver nanoparticles did not modify the osteoconductive properties, cell attachment, or proliferation of bone cells. In contrast, it provided favorable macrophage inflammatory responses, offering both osteo-immunomodulatory and antibacterial effects. Similar results were reported by Iffat Jahan et al. [61] using silver/GelMA as a scaffold for wound healing, where silver/GelMA exhibited antibacterial activity against both Gram-positive and Gram-negative bacterial strains without affecting cell viability.

### 4.3. Inorganics-Based Nanomaterials

In general, GelMA provides a structure similar to the ECM for cells to proliferate. However, besides the basic requirement like a substrate for cell attachment, the supplemental composition should also be considered to provide an optimal environment for different cell types. Inorganic materials can add minerals to GelMA to better resemble natural ECM [121]. A series of inorganic materials such as hydroxyapatite (HAp) or bioglass, silicate, ceramics, or other porous nanoparticles have been used to modify GelMA.

GelMA can be functionalized with HAp, a type of calcium phosphate generally found in bone that promotes the formation of new bone. It has been shown that a higher concentration of HAp in the presence of VEGF enhanced bone regeneration [62], as observed in Figure 6c. Su et al. [63] showed that a composite hydrogel based on silanized GelMA/HAp enhanced the mechanical properties of the hydrogel and displayed high biocompatibility for bone tissue engineering. A major limitation of HAp is its poor biodegradation. Biphasic calcium phosphate (BCP) is a combination of HAp and beta-tricalcium phosphate that is stable, rigid, and biodegradable, which is suitable for bone tissue engineering [64].

Another material with great osteoconductivity and biodegradability is bioglass (BG). BG is a silicate-based commercial bioactive material with properties suitable for bone regeneration. BG is fragile by itself and does not have the flexibility to maintain its own structure. To improve the mechanical strength of BG, Kwon et al. [123] incorporated GelMA and reported that BG was able to generate HAp on its surface in a bone regeneration model. Recent studies have also utilized BG modified GelMA hydrogel to stabilize the release of osteogenic growth factors and other proteins. For example, rhBMP-2 is well-known for treating bone defects, but rhBMP-2 offers an uncontrolled local concentration that causes undesired side effects. Xin et al. [65] combined BG and rhBMP-2 to form a GelMA/MBGNs-rhBMP-2 hydrogel membrane and showed promising results to simulate periosteum.

The presence of MBGNs-rhBMP-2 favored differentiation of mesenchymal stem cells for longer periods of time. Zhang et al. [66] photo-crosslinked GelMA and quaternized chitosan with Mg^2+^ to prepare magnesium-based polyhedral oligomeric silsesquioxane (POSS) nanoparticles. POSS serves as an inorganic crosslinker that can be incorporated into an organic polymer matrix. Following this principle, his technique made the material resilient and tough and facilitated in situ bone regeneration [39]. Clay is another nanomaterial commonly studied for biomedical applications. When combined with gelatin, clay is able to functionalize a variety of polymers. Laponite is a commercially available clay nanomaterial that has been combined with gels to produce new bioink or drug carriers. Similar to BG, clay is able to bind proteins in their structure, offering not only osteogenic properties but also adding an angiogenic effect. Cidonio et al. [67] manufactured a nanocomposite using laponite and GelMA as bioink. As a scaffold, this newly developed bioink showed excellent shape retention and allowed tunable viscoelastic properties that lasted for 21 days in vitro.

## 5. Biomedical Applications

Tissue engineering aims to assemble functional constructs to restore damaged tissues from trauma, disease, or aging. In the past 20 years, the fabrication of biomaterials and production of temporary scaffolds have shown promising results both in vitro and in vivo. GelMA has very diverse biomedical applications in its manufacturing process depending on the purpose of the material. For example, GelMA can be described as a hybrid bioink for bone regeneration or applied as a skin dressing, which both utilize bioprinting as the manufacturing process [39]. As different tissues, such as the bone, skin, muscle, and brain, have very different environments surrounding their cells, the bioink composition needs to be tuned to mimic each specific environment. In the following section, we will discuss the biomedical applications of GelMA from a tissue-specific perspective following different manufacturing processes.

### 5.1. Neural Tissues

The nervous system consists of the central nervous system (CNS) and the peripheral nervous system (PNS). The brain, spinal cords, and a few cranial nerves (optical, olfactory, auditory) belong to the CNS, while the remaining cranial nerves, spinal cords, and sensory nerves that interact with muscle or skin are considered the PNS. The CNS is composed primarily of neurons that can only regenerate portions under certain conditions, while the PNS consists mainly of glial cells that have capacities of cell division. Therefore, most studies have targeted PNS injuries. Currently, the clinical approach for peripheral nerve injury is either a direct end–end surgical connection or an autologous graft [124]. Therefore, the tissue engineering approach has been to 3D print a complex GelMA hydrogel scaffold for nerve regeneration. XMU-MP-1, an inhibitor of the Hippo pathway known to regulate organ size, tissue size, and tumorigenesis, has been shown to promote more efficient regeneration in the study by Tao et al. [68], where they functionalized XMU-MP-1 using GelMA and 3D printed a drug-releasing conduit. Three months after removing 5 mm of the sciatic nerve in rats and using the conduit as a bridge to connect both ends, they observed stable guidance for neurite extension. This indicates a promising alternative to autograft for peripheral nerve repair [68]. Another study used a GelMA tube loaded with microspheres containing glial cells and growth factors for attempted nerve regeneration.

Although the proximal stump commenced a regeneration process, the distal underwent degeneration [69]. Other studies have reported designs with multiple combinations and architectural structures, such as a 3D-printed multichannel conduit by Ye et al. [125] and GelMA with added carbon nanotubes by Park et al. [54]. Regeneration of the CNS remains challenging because of its insufficient regeneration capacity and cerebrospinal fluid surroundings. For CNS regeneration, natural polymers provide good biocompatibility but lack mechanical strength. Chen et al. [126] aligned GelMA into fibrous bundles using parallel modified-electrode rods to guide neuronal cell migration or axon extension. The group showed that the material promoted cell migration and functionality in vitro with the unaddressed challenges of having a functional vascular system or neuronal stimulus. Other researchers used GelMA functionalized by dopamine as a scaffold to induce neuronal stem cell differentiation and showed a visible neural network formed within the scaffold after 12 days. This indicates that custom-made scaffolds can promote differentiation [70]. A similar finding was reported in a rat brain injury model treated with injectable GelMA hydrogel carrying human amniotic mesenchymal stromal cells and imidazole. Imidazole has been observed to promote the differentiation of stem cells into neural cells. The results showed that the treated group had the smallest injured area compared to controls, indicating a possible new treatment option for brain regeneration [71].

### 5.2. Bone

An ideal scaffold should (1) provide biocompatibility, (2) promote cell adhesion and proliferation, (3) mimic the properties of the natural environment with a porous architecture that allows for cell proliferation and vascularization, and (4) match a degradation rate that compares to the formation rate of the new tissue [127]. One of the objectives for bone regeneration is to repair lost or damaged bone at an appropriate rate. Bone tissues contain inorganic minerals and multiple cell types with an extracellular matrix of collagen fibrils and carbonated hydroxyapatite in between. Together, these components offer bones excellent mechanical and chemical properties. Similar to neuronal tissue, current clinical treatments for bone injury are mainly bone autographs and allographs. Bone regeneration can be achieved through an artificial scaffold to allow migration and differentiation of cells until the healing process has finished. Importantly, aging is an essential factor to consider in this approach as aged bones become fragile and lose their mechanical properties [128]. One of the most significant challenges of bone regeneration is to strike a balance between irrigation and rigidity of the structure while maintaining a consistent degeneration rate of the material to allow cells to form a natural structure.

Recently, great attention has been placed on endochondral bone regeneration, when cartilage intermediaries are replaced by mature bone structures. This process naturally occurs during birth, as the bone turns from soft cartilaginous tissue to a rigid and strong bony structure. Following this concept, Daly et al. [129] used a sacrificial Pluronic ink to 3D print patterned and interconnected microchannels inside the GelMA hydrogel. They implanted the structure seeded with mesenchymal stem cells inside for femurs in rats and showed an improvement in vascular formation. In addition, the implantation of cartilage following an endochondral approach developed a cascade of events, such as the expression of the angiogenic factor of VEGF, that mimicked bone development in vivo. GelMA hydrogels can also be injected into a weak or injured bone to enhance its regeneration. To increase the number of drugs compatible with GelMA for hydrogel doping, liposomes have been used as a carrier to contain specific drugs. Cheng et al. [130] reported double crosslinked-GelMA hydrogel enhanced by liposomes containing deferoxamine (DFO), paclitaxel (PTX), and bovine serum albumin (BSA). Due to the different processes involved in bone regeneration, this procedure must be tuned as a phased release that can extend the effect of the drugs. Other studies have also reported the use of GelMA hydrogel for cartilage [72] and tendon [44] formation.

### 5.3. Vasculature

Blood vessels are the primary means to deliver nutrients and oxygen to tissues in the body. Vascular disorders are among the top causes of death worldwide. In the current state of tissue engineering, vascularization remains a primary challenge as blood vessels must be able to withstand a wide range of blood pressures and offer a range of sizes, including small vessels with 1–6mm diameters. Several approaches have been investigated, such as applications of cytokines, genes, or cell differentiation to stimulate vascular formation [131]. Currently, designing a scaffold to guide cells into vascular differentiation is an attractive approach for vascular regeneration. GelMA hydrogels have excellent mechanical properties as vascular scaffolds. However, obtaining a large vascular network that can dive deep into tissue has not been possible. Shao et al. [132] used a co-axial bioprinting method to generate GelMA microfibers. The hydrogel shape resembled a human umbilical cord with human umbilical cord endothelial cells encapsulated in the fiber. The encapsulated cells followed a migration pattern towards the peripheries of the fiber and formed a lumen structure that could potentially be used for other applications.

Recently developed printing techniques can precisely create microfluidic devices. Digital micromirror device-based projection printing is a continuous digital light processing printing technique that offers a high throughput and great biocompatibility for cell seeding and encapsulation. Miri et al. used PEGDA or GelMA to bioprint multiple structures with a printing resolution of 20–30 µm using 2–3 different bioinks. The structures were reported to lead to more blood vessel formation in the presence of VEGF [73]. Cells can successfully differentiate when seeded in a suitable environment and exposed to multiple growth factors. GelMA hydrogels can easily provide modified structural properties with different concentrations and dope multiple agents within their structure, mimicking a natural condition that can benefit tissue cultures [133]. Other approaches include generating a morphing scaffold that can convert from a 2D planar shape into a 3D tubular shape to help with vessel formation. Zhao et al. used poly-ε-caprolactone and GelMA to favor a homogeneous endothelial cell layer with reshaping capabilities and showed maintained cell–cell interaction [74].

### 5.4. Muscle

Muscles are contractile tissues responsible for nearly all movement in the body. Its configuration consists of fibers arranged in a parallel order, with each fiber being a long cylindrical multinuclear cell surrounded by connective tissues. Muscles are one of the most highly vascularized organs. Muscle cells work together in an orchestrated pattern to provide strength, and this is an essential factor to consider while designing their scaffold [134]. It has been reported that 3D-bioprinted scaffolds can improve the cell distribution and configuration of the muscle tissue. Seyedmahmoud et al. [135] showed that 10% (*w*/*v*) GelMA with 8% (*w*/*v*) alginate and added CaCl_2_ provided the optimal condition for muscles. For clinical applications, a substitute of muscle must be able to sustain and respond to electrical stimulation. Want et al. [136] enhanced myogenic differentiation by designing a scaffold based on poly-3,4-ethylene dioxythiophene, a conductive material deposited in GelMA. The ECM-like environment favored the growth of C2C12 cells encapsulated in the fabricated hydrogel. Nanoparticle modifications and cell exposure to an electrical field have both been studied to improve conductivities [137]. To improve cell alignment, acoustic cell patterning with 3D bioprinting techniques and ultrasound waves have been used to manipulate cell populations within the biomaterial dynamically [134]. Ostrovidov et al. [138] used a micropatterned GelMA hydrogel to improve cell alignment by co-culturing myoblasts and neural cells. Interestingly, their observation showed that the presence of neural cells improved muscle cell differentiation and myotube formation.

### 5.5. Skin

Skin is the largest organ in the human body. Its complicated structure allows us to have a semipermeable protective barrier with important functions that help to maintain internal homeostasis. It is composed of keratinocytes that form the outside protective layer, melanocytes that provide skin color, and fibroblasts that offer strength and elasticity. Skin cells have a valuable regeneration rate that is able to heal wounds of up to 4 cm in diameter [139]. For larger and deeper wounds, factors such as vascularization and sterilization can significantly affect the regeneration process. Therefore, larger skin damage will need natural or artificial skin grafts to help with healing. Numerous types of artificial skin have been developed, but challenges such as safety, cost, and angiogenesis remain. Biomedical applications of GelMA hydrogel for skin tissue engineering range from strong microneedle wound dressings to 3D-bioprinted artificial skin grafts for transplantation [139]. Recently, Luo et al. [140] tested a GelMA hydrogel loaded with doxorubicin as a transdermal drug patch and showed that the constructs were strong enough to penetrate the skin and deliver the drug through both swelling and enzymatic degradation. Insoluble drugs such as cyclodextrin have also been delivered through the hydrogel approach [141]. Other GelMA microneedle applications have been used as a noninvasive technique to extract interstitial fluid from the skin to detect biomarkers [142]. When skin loses its protective function, it becomes susceptible to infections. In general, clinicians need to use antibiotics as preventive measures to keep a large wound clean. Huang et al. [120] embodied graphene and nitric oxide to create a wound dressing compatible with photothermal therapy that improved the wound healing process. Liu et al. [143] designed a dressing that was functional underwater aimed at less sutured skin and applications in stomach surgery. For skin transplantation, one of the major challenges is the immediate availability of transplantable tissue. It is also not possible to generate large portions of engineered artificial skin to use in clinical settings. Zhou et al. [144] used digital light processing 3D printing to produce a functional living skin using GelMA hydrogel with butanamide-linked hyaluronic acid and lithium phenyl-2,4,6-trimethylbenzoylphosphinate as photoinitiators and showed superior performance in skin regeneration. Jin et al. [145] printed a PEG-based acellular dermal matrix and a GelMA based structure to provide cells with a natural microenvironment for improved re-epithelization and angiogenesis. To induce a vascularized network, Turner et al. [146] designed a bioink consisting of 13% (*w*/*v*) cell-laden GelMA with a core-shell composed of chitosan and dextran aldehyde. This composition contained bone marrow mesenchymal stem cells and umbilical vein endothelial cells inside the core. Microvascularization was observed in a single-step procedure, indicating that they were able to produce a safe vehicle for cells that might facilitate improvements for wound healing. Other approaches include the use of HUVECs in GelMA hydrogel to maintain a sustained release of exosomes to promote skin regeneration and vascularization [147]. Many growth factors proved to improve vascularization but suffered from enzymatic degradation of the GelMA hydrogel that offered a protective barrier to prolong their effect. Li et al. [148] functionalized endothelin-1 and observed full thickness and better blood vessel formation after 14 days in rat models.

## 6. Conclusions and Perspectives

In the past 20 years, there have been tremendous advancements in the field of tissue engineering. Yet, much remains unknown. The complexity of reproducing a small portion of tissue still eludes our complete understanding. However, the puzzle is filling in. First, we now know that the precise characteristics of the scaffold are essential for the development of tissue. Although GelMA by itself is an excellent biomaterial for tissue culture, achieving better results requires specific modifications for the particular tissue it is designed to support. Oftentimes, doping GelMA with a series of agents can improve its regenerative properties. Second, we have realized how critical it is to create a cell microenvironment similar to that found in humans. More studies are needed to identify further the best conditions for growing specific tissues. One of the most significant challenges—and opportunities—is generating a functional vascular system that can increase the viability of the tissue and allow the co-culture of multiple cells that can interact with each other. Breakthroughs in these areas will help to improve our understanding of the interaction between tissues and get us much closer to regenerating artificial organs. Advances have been made for specific tissues, such as bones, that are inching closer to biomedical applications in the near future.

## Figures and Tables

**Table 1:** Summary of GelMA with different modifiers for tissue engineering applications.

Modifier	Optimal Modifier Concentration	Optimal GelMA Concentration	GelMA Crosslinking Condition	Application of Hydrogel	Refs.
VEGF	400 ng/mL	9 *w*/*v*%	0.067 *w*/*v*% LAP, 395 nm UV, 20 s	Wound healing in a porcine model	[23]
VEGF-mimicked peptide	200 μg/mL	5 *w*/*v*%	0.5 *w*/*v*% Irgacure 2959, 7 mW/cm^2^ UV, 30 s	In vitro microvascularization	[24]
	100 μg/mL	2 mg	0.0002 *w*/*v*% Irgacure 2959, 365 nm UV, 20 min	In vitro microvascularization	[25]
	0.02 *w*/*v*%	15 *w*/*v*%	1 *w*/*v*% Irgacure 2959, 365 nm UV, 5 min	Wound healing in a porcine model	[26]
AAV-VEGF	1 × 10^10^ vg	20 *w*/*v*%	0.5 *w*/*v*% Irgacure 2959, 350 mW/cm^2^ UV, 60 s	Ischemic stroke therapy in a rat model	[27]
TGF-β3	10 ng/mL	10 *w*/*v*%	0.3 *w*/*v*% LAP, 405 nm, 30 s	Cartilage regeneration in a rat model	[28]
	240 ng/construct	10 *w*/*v*%	0.05 *w*/*v*% Irgacure 2959, 365 nm UV, 15 min	Sustain TGF-β3 supply for chondrogenesis	[29]
TGF-β1-affinity peptide	0.025 mM	20 *w*/*v*%	365 nm UV light, 5 min	Chondrogenesis and cartilage regeneration	[30]
Platelet lysates	50 *v*/*v*%	7.5 *w*/*v*%	0. 0.375% *w*/*v*% LAP, 405 nm	Angiogenesis in endodontic regeneration	[31]
	50 *v*/*v*%	15 *w*/*v*%	0.06 *w*/*v*% LAP, 400 nm UV, 60 s	Skin tissue engineering in a mouse model	[32]
Hyaluronic acid	1 *w*/*v*%	15 *w*/*v*%	0.3 *w*/*v*% Irgacure 2959, 365 nm UV, 40 s	Vascularized dermis	[33]
	5 *w*/*v*%	7 *w*/*v*%	0.03 *w*/*v*% LAP, UV, 20 s	Cartilage regeneration	[34]
	5 *w*/*v*%	15 *w*/*v*%	365 nm UV, 5 min	Skin wound healing	[35]
Chitosan	0.5 and 1 *w*/*v*%	10 *w*/*v*%	0.1 *w*/*v*% Irgacure 2959, 365 nm UV, 30 s, 0.1 M NaOH 30 min	Semi- and full-interpenetration network hydrogel	[36]
	1 *w*/*v*%	5 *w*/*v*%	0.3 *w*/*v*% Irgacure 2959, 365 nm UV, 60 s, 0.3 M NaOH 30 s	In vitro 3D neurite outgrowth and elongation	[37]
	1 *w*/*v*%	3 *w*/*v*%	0.25 *w*/*v*% Irgacure 2959, 365 nm UV, 1–3 min	Thermo-responsive contraction scaffold for 3D culture	[38]
	3 *w*/*v*%	15 *w*/*v*%	Irgacure 2959, 365 nm UV, 5 min	Bone osteogenesis and angiogenesis in a rat model	[39]
	2.5 *w*/*v*%	2 *w*/*v*%	Ascorbic acid and H_2_O_2_ at 37 °C, ≤60 s	Antibacterial wound closure in a rat model	[40]
Alginate	1 *w*/*v*%	1 *w*/*v*%	0.25 *w*/*v*% Irgacure 2959, UV	Wound healing and soft tissue regeneration	[41]
	1 *w*/*v*%	10 *w*/*v*%	1 *w*/*v*% Irgacure 2959, UV, 30 s	Bioprinting blood vessels	[42]
	5 *w*/*v*%	5 wt%	0.5 wt% Omnirad 2959, 365 nm UV, 10 min	Antibacterial and bone repair in a mouse model	[43]
	4 *w*/*v*%	5 *w*/*v*%	0.1 *w*/*v*% Irgacure 2959, 365 nm UV, 30 s, 0.1 M NaOH 30 min	Novel bioink	[44]
Silk fibroin	10 *w*/*v*%	10 *w*/*v*%	0.1 *w*/*v*% LAP, 365 nm UV, 2 min	Wound healing in a mouse model	[45]
	1 *w*/*v*%	10 *w*/*v*%	0.1 mM eosin Y, 0.2 *w*/*v*% NVP 0.2 *w*/*v*% TEA, visible light	Novel bioink	[46]
	5.6 *w*/*v*%	2.4 *w*/*v*%	0.1 *w*/*v*% Irgacure 2959, UV 30 s	Encapsulate cells or growth factors	[47]
	8 wt%	10 wt%	0.5 *w*/*v*% Irgacure 2959, 365 nm UV, 3 min	Corneal regeneration	[48]
	8 wt%	10 wt%	1 *w*/*v*% Irgacure 2959, 365 nm UV, 30 s	Stroma tissue regeneration	[49]
CNT	0.3 *w*/*v*%	7 *w*/*v*%	0.5 *w*/*v*% Irgacure 2959, UV, 50 s	The construction of functional engineered cardiac tissues	[50]
	0.01 *w*/*v*%	N/A	0.5 *w*/*v*% Irgacure 2959, UV, 50 s	Cardiac regeneration	[51]
rGO	0.05 *w*/*v*%	15 *w*/*v*%	UV irradiation	Heart-on-a-chip system with dynamic self-reporting function	[52]
	0.1 *w*/*v*%	6 *w*/*v*%	0.5 *w*/*v*% LAP, 405 nm UV, 60 s	Conductive scaffold for cardiac microtissue maturation	[53]
	0.2 *w*/*v*%	20 *w*/*v*%	2% *w*/*v* APS, 60 °C, 6 h	Nerve guidance conduits in a rat sciatic nerve defect model	[54]
ND	0.2 *w*/*v*%	7 *w*/*v*%	0.1 *w*/*v*% Irgacure 2959, UV, 6 min	Bone regeneration	[55]
rGO	0.05 *w*/*v*%	15 *w*/*v*%	UV irradiation	Heart-on-a-chip system with dynamic self-reporting function	[52]
	0.1 *w*/*v*%	6 *w*/*v*%	0.5 *w*/*v*% LAP, 405 nm UV, 60 s	Conductive scaffold for cardiac microtissue maturation	[53]
SiGO	0.142 *w*/*v*%	15 *w*/*v*%	0.15 *w*/*v*% LAP, 395 nm UV	Osteogenesis of human mesenchymal stem cells	[56]
Gold nanorod	0.01 *w*/*v*%	7 *w*/*v*%	0.25 *w*/*v*% Irgacure 2959, UV, 30 s	Print 3D cardiac constructs	[57]
	1.5 *w*/*v*%	5 *w*/*v*%	0.5 *w*/*v*% Irgacure 2959, UV, 30 s	Maturation and functionalities of the cardiac tissue	[58]
Gold nanowires	0.03 *w*/*v*%	10 *w*/*v*%	0.5 *w*/*v*% LAP, 405 nm UV, 32 s	Construct functional cardiac tissue	[59]
Nanosilver	2 mM	15 *w*/*v*%	0.1 *w*/*v*% Irgacure 2959, 365 nm UV, 30 s	Treatment for infected bone defects	[60]
AgNP	50 μg/ml	15 *w*/*v*%	1.5 *w*/*v*% Irgacure 2959, 365 nm UV, 60 s	Antibacterial activity for prosthesis	[61]
HAp	0.2 *w*/*v*%	5 *w*/*v*%	1 *w*/*v*% Irgacure 2959, UV, 5 min	In vitro capillary formation for bone tissue engineering	[62]
Si-HAp	3 *w*/*v*%	15 *w*/*v*%	1 *w*/*v*% VA-086, 440 nm blue light, 1 min	Enhanced the mechanical properties of the composite hydrogel	[63]
BCP	10 mg	15 *w*/*v*%	0.05 mM eosin Y, 1.88 *v*/*v*% TEA, 1.25 *w*/*v*% ascorbic acid, visible light visible light 120 s	Cell viability and relatively bone differentiation ability	[64]
MBGNs	3 *w*/*v*%	5 *w*/*v*%	1 *w*/*v*% Irgacure 2959, UV, 3 min	Simulate the periosteum and promote bone reconstruction	[65]
QSC	3 *w*/*v*%	20 *w*/*v*%	0.5 *w*/*v*% Irgacure 2959, 365 nm UV, 10 min	Bone repair with antibacterial properties	[66]
Laponite	1 wt%	7.5 wt%	1 mM [Ru(dmbpy)_3_](PF_6_)_2_, 10 mM SPS, visible light, 3 min	Osteogenic and angiogenic tissue formation	[67]
MPEG-PCL	49.5 *w*/*v*%	20 *w*/*v*%	visible light (405 nm)	Recovery of peripheral nerve injuries	[68]
GDNF-loaded microspheres	8 μg/ml	15 *w*/*v*%	200 mM EDC, 10 h	Sciatic nerve growth	[69]
Dopamine	30 *w*/*v*%	6.25 *w*/*v*%	1 *w*/*v*% Irgacure 2959, 355 nm UV	Neural regeneration	[70]
Polydopamine@SDF-1α	0.1 *w*/*v*%	5 *w*/*v*%	0.1 *w*/*v*% LAP, 405 nm UV, 30 s	Stem cell differentiation and repair of focal brain injury	[71]
PEGDA	5 *w*/*v*%	10 *w*/*v*%	1 *w*/*v*% Irgacure 2959, 355 nm UV, 45 s	Cartilage regeneration	[72]
	50 *v*/*v*%	7 *w*/*v*%	0.3 *w*/*v*% LAP, 365 nm UV	Novel bioink	[73]
PCL	5 *w*/*v*%	5 *w*/*v*%	Irgacure 2959, UV, 15 min	3D endothelialization	[74]

N/A—data not available.

## Data Availability

Not applicable.
